# Effects of exercise interventions on gait, motor function, and balance in patients with Parkinson’s disease: A meta-analysis with clinical implications

**DOI:** 10.1371/journal.pone.0359274

**Published:** 2026-09-25

**Authors:** Chengshuo Zhang, Zhen Jia, Yongdi Wang, Wanli Zang

**Affiliations:** 1 Henan University of Science and Technology, Luoyang, Henan, China; 2 Guangdong Polytechnic of Science and Technology, Zhuhai, Guangdong, China; 3 School of Physical Education, Soochow University, Suzhou, Jiangsu, China; Mae Fah Luang University School of Anti Aging and Regenerative Medicine, THAILAND

## Abstract

**Objective:**

Evidence regarding the effects of exercise interventions on gait-related symptoms, motor function, and balance in patients with Parkinson’s disease (PD) remains uncertain across outcome measures and intervention characteristics. This study aimed to evaluate the effects of exercise interventions on the Freezing of Gait Questionnaire (FOGQ), Unified Parkinson’s Disease Rating Scale Part III (UPDRS-III), Berg Balance Scale (BBS), and Mini-Balance Evaluation Systems Test (Mini-BESTest), and to explore potential sources of heterogeneity.

**Methods:**

Standardized mean differences (SMDs; Hedges’ g) with 95% confidence intervals (CIs) were pooled using random-effects restricted maximum likelihood models. Exploratory subgroup analyses examined age, disease duration, intervention duration, training frequency, single-session duration, and exercise modality as potential sources of heterogeneity. Risk of bias was assessed using the Cochrane Risk of Bias 2 tool, and certainty of evidence was assessed using Grading of Recommendations Assessment, Development and Evaluation. This review was registered in PROSPERO (CRD420261281718).

**Results:**

The meta-analysis included 20 randomized controlled trials published between 2009 and 2024, involving 714 patients with PD, including 372 participants in the exercise intervention groups and 342 in the control groups. Sixteen trials were judged as having some concerns and four as having a high overall risk of bias; none was rated as low risk. Compared with control conditions, exercise interventions were associated with lower FOGQ scores (SMD = −0.62, 95% CI −1.06 to −0.18) and UPDRS-III scores (SMD = −0.80, 95% CI −1.02 to −0.59), and higher BBS scores (SMD = 0.85, 95% CI 0.27 to 1.42). The pooled effect for Mini-BESTest was not statistically significant (SMD = 0.23, 95% CI −0.24 to 0.70). Exploratory subgroup analyses did not establish differential effects according to exercise modality, dose parameters, age, or disease duration. The certainty of evidence was moderate for UPDRS-III and low for FOGQ, BBS, and Mini-BESTest.

**Conclusion:**

Exercise interventions may improve freezing-of-gait symptoms, overall motor function, and some aspects of balance performance in patients with PD, although evidence for Mini-BESTest outcomes remains inconclusive. Given the limited certainty of evidence and methodological limitations of the included trials, these findings warrant cautious interpretation.

## Introduction

Parkinson’s disease (PD) is a progressive neurodegenerative disorder characterized by the degeneration of dopaminergic neurons in the substantia nigra and the deposition of Lewy bodies [1]. With the accelerating aging of the global population, the burden of PD continues to increase. In 2021, there were approximately 11.9 million patients worldwide, and it is projected to rise to about 25.2 million by 2050 [2]. Besides the typical symptoms such as resting tremor, bradykinesia and rigidity, PD patients often experience gait abnormalities, impaired postural control and an increased risk of falls [3,4], which significantly affect their mobility, independence in daily life and quality of life. Therefore, rehabilitation strategies targeting gait impairment, postural instability, and motor dysfunction are clinically important in the comprehensive management of PD [5].

Although drug therapy remains the fundamental treatment strategy for PD, its effects on gait impairment, balance dysfunction, and postural instability are often limited, and long-term application may be accompanied by end-of-dose phenomenon, dyskinesia and symptom fluctuations, etc. [6,7]. In contrast, exercise interventions have advantages such as a favorable safety profile, long-term feasibility, and broad applicability [8], and can provide support for motor function recovery by improving lower limb muscle strength, walking ability and balance control [9]. Therefore, exercise intervention has become an important part of PD functional rehabilitation.

Previous systematic reviews and meta-analyses have evaluated the efficacy of exercise therapy or physical therapy in patients with PD, but there are still deficiencies. Some studies have focused on a single exercise modality or specific training domains, which limits the integration of evidence from different intervention strategies [10]. At the same time, existing reviews lack systematic analysis of dose parameters such as intervention duration, training frequency, and duration of each training session, making it difficult to generate hypotheses for future research on exercise prescription [11]. In addition, the outcome measures used in different studies are not consistent, and different scales have differences in measurement dimensions and psychometric properties, which may also affect effect estimation and evidence interpretation [12].

Accordingly, this systematic review and meta-analysis aimed to evaluate the effects of exercise interventions on gait-related symptoms, overall motor function, and balance in people with PD. The principal outcomes were the Freezing of Gait Questionnaire (FOGQ), Unified Parkinson’s Disease Rating Scale Part III (UPDRS-III), Berg Balance Scale (BBS), and Mini-Balance Evaluation Systems Test (Mini-BESTest). We also explored potential sources of heterogeneity and assessed the certainty of evidence for each outcome.

## Materials and methods

### Study framework

This systematic review and meta-analysis was conducted in accordance with the Preferred Reporting Items for Systematic Reviews and Meta-Analyses (PRISMA) 2020 statement and was prospectively registered in PROSPERO (CRD420261281718) [13]. The population, intervention, comparator, outcomes, and study design (PICOS) criteria are presented in Table 1.

**Table 1:** PICOS criteria for study inclusion.

Population	Intervention	Comparator	Outcome	Study design
Patients with PD	Intervention setting: rehabilitation centres, hospitals	usual care	FOGQ, UPDRS-III	Randomized controlled trials (RCTs)
Age ≥ 18 years	Intervention providers: rehabilitation therapists	usual rehabilitation	BBS, Mini-BESTest	
	Intervention type: exercise-based interventions, including single-modality exercise and combined/multimodal training	no structured exercise		
	Intervention parameters: intervention duration, intervention frequency, single-session intervention time			

Ethics approval and consent to participate were not applicable because this study was based on previously published studies and did not involve direct recruitment of human participants or collection of identifiable personal data.

### Literature search strategy

We systematically searched PubMed, Embase, Web of Science, Cochrane Central Register of Controlled Trials (CENTRAL), and Scopus from the establishment of each database until January 10, 2026. The search terms included Parkinson’s disease, exercise intervention, and randomized controlled trials. The complete search strategies for each database, including database platform, exact search terms, search date, restrictions, language limits, and manual and citation-search methods, are provided in Appendix 1. In addition, we conducted a backward search of the references of the included studies and related reviews, and supplemented with necessary manual searches.

### Eligibility criteria

Inclusion criteria (PICOS)**:** (P) adults aged ≥18 years with idiopathic PD diagnosed according to accepted clinical criteria (diagnostic criteria and staging were as reported in the original studies), with medication status/stability reported where available; (I) the intervention group received exercise-only interventions, defined as structured physical training without additional non-exercise therapeutic components as the active experimental element; in multi-arm trials that included both an exercise-only arm and a combined-intervention arm, eligibility was determined at the arm level, and only the separately extractable exercise-only arm was eligible for inclusion; (C) the control group received usual care/usual rehabilitation or no structured exercise intervention; (O) at least one of the following outcomes was reported: FOGQ, UPDRS-III, BBS, or Mini-BESTest; (S) randomized controlled trials (RCTs).

Exclusion criteria: (1) motor/gait impairment due to other neurological disorders (e.g., stroke, multiple sclerosis, dementia); (2) interventions including non-exercise components or combined treatments that could not be disentangled from the exercise effect (e.g., medication adjustment, electrical stimulation, cognitive training, or music therapy); studies with mixed interventions were excluded unless data for an exercise-only arm could be extracted separately; (3) missing key data, non-convertible statistics, or inability to extract effect sizes; (4) duplicate publications (retaining the most complete report or the one with the largest sample size); (5) abstract-only reports or unavailable full texts (e.g., conference abstracts, registration records only).

### Study selection and data extraction

All retrieved records were imported into EndNote X20 for management, and duplicates were removed by software and manual checking. Two reviewers independently screened titles, abstracts, and full texts according to the prespecified eligibility criteria. Disagreements were resolved by discussion or by a third reviewer. Reasons for full-text exclusion were recorded and summarized in the PRISMA 2020 flow diagram.

Data were extracted independently by two reviewers using a standardized form. Extracted information included study characteristics, participant characteristics, intervention and control details, exercise dose, outcome data for FOGQ, UPDRS-III, BBS, and Mini-BESTest, adverse events, and information required for risk-of-bias assessment. For each outcome, data measured immediately after completion of the intervention were extracted for the primary meta-analysis; follow-up data were not pooled because follow-up timing and reporting were inconsistent across trials.

### Methodological quality assessment

The Cochrane Risk of Bias 2 (RoB 2) tool was used to assess risk of bias in the included RCTs [14]. The RoB 2 assessment comprised five bias domains and an overall risk-of-bias judgment: (1) bias arising from the randomization process, (2) bias due to deviations from intended interventions, (3) bias due to missing outcome data, (4) bias in measurement of the outcome, (5) bias in selection of the reported result, and (6) overall risk of bias. Each component was judged as “low risk of bias”, “some concerns”, or “high risk of bias”. Two reviewers independently assessed the included studies, and disagreements were resolved through discussion or consultation with a third reviewer.

### Statistical analysis

Meta-analyses were conducted using Stata 17.0. Continuous outcomes were pooled as standardized mean differences (SMDs, Hedges’ g) with 95% confidence intervals (CIs). Random-effects restricted maximum likelihood (REML) models were used for all analyses. Heterogeneity was assessed using Cochran’s Q test and quantified using I^2^ and τ^2^, and the heterogeneity results were used for interpretation rather than model selection [15]. Prediction intervals were considered for outcomes with moderate or high heterogeneity; however, they were not reported because the number of included studies for these outcomes was limited, which could make prediction interval estimates unstable and potentially misleading.

Prespecified subgroup analyses were conducted according to age, disease duration, intervention duration, training frequency, single-session duration, and exercise modality. Sensitivity analyses were performed using leave-one-out analyses. Publication bias and small-study effects were assessed using funnel plots and Egger’s regression test [16]. Formal assessment was performed for outcomes with at least 10 comparisons, whereas outcomes with fewer than 10 studies were interpreted cautiously. A two-sided P < 0.05 was considered statistically significant.

### Certainty of evidence assessment

The certainty of evidence for each primary outcome was assessed using the Grading of Recommendations Assessment, Development and Evaluation (GRADE) approach, considering risk of bias, inconsistency, indirectness, imprecision, and publication bias [17]. Evidence certainty was classified as high, moderate, low, or very low, and the results are presented in the Summary of Findings table.

## Results

**Fig 1:**
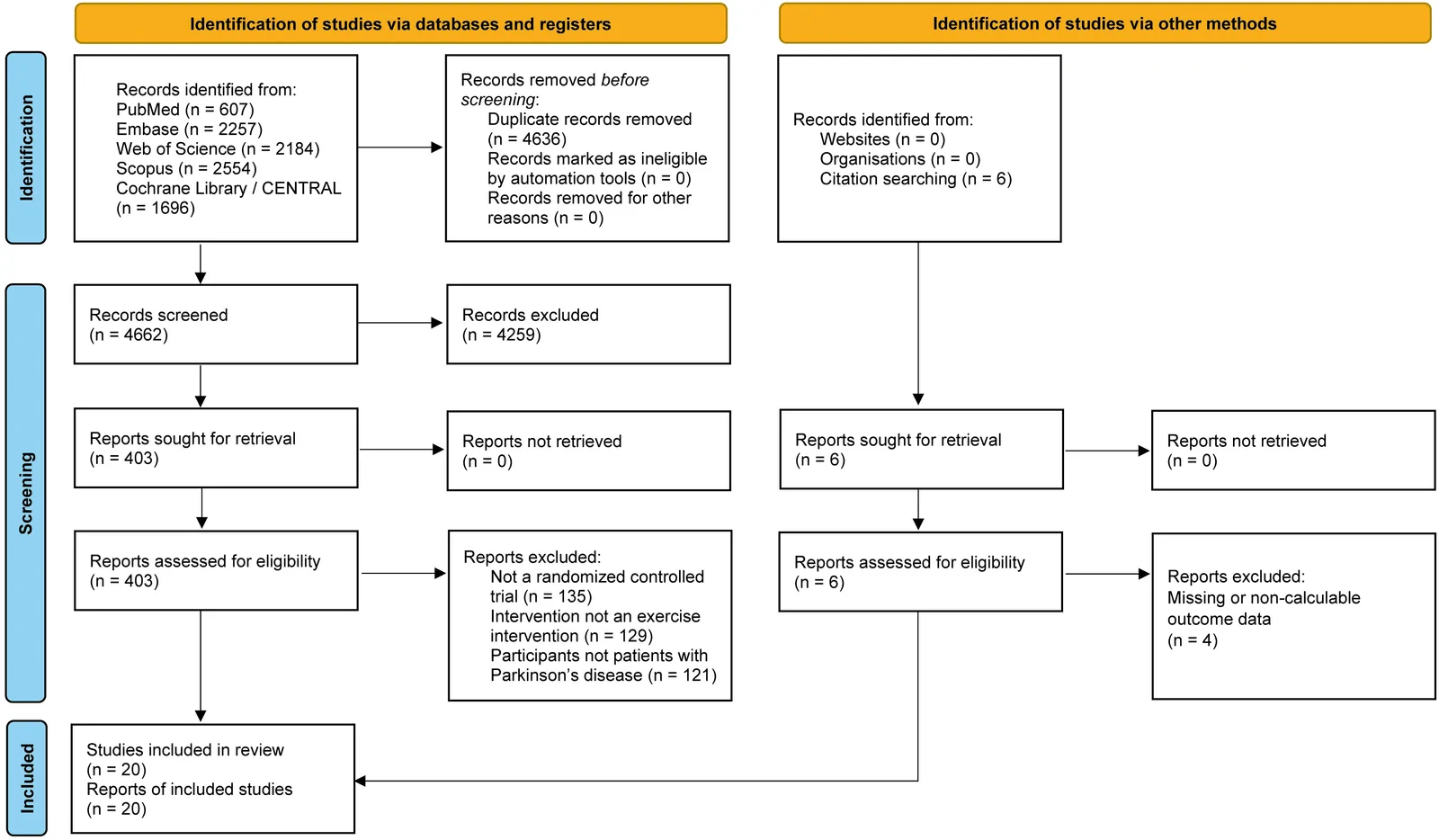
PRISMA 2020 flow diagram of literature search and study selection.

Database searches identified 9,298 records. After 4,636 duplicate records were removed, 4,662 records underwent title and abstract screening, of which 4,259 were excluded. The remaining 403 reports were retrieved and assessed for eligibility. Citation searching identified six additional reports, all of which were retrieved and assessed for eligibility. Thus, a total of 409 full-text reports were assessed, of which 389 were excluded. Finally, 20 RCTs met the eligibility criteria and were included in the meta-analysis, as shown in Fig 1.

### Risk of bias in included studies

**Fig 2:**
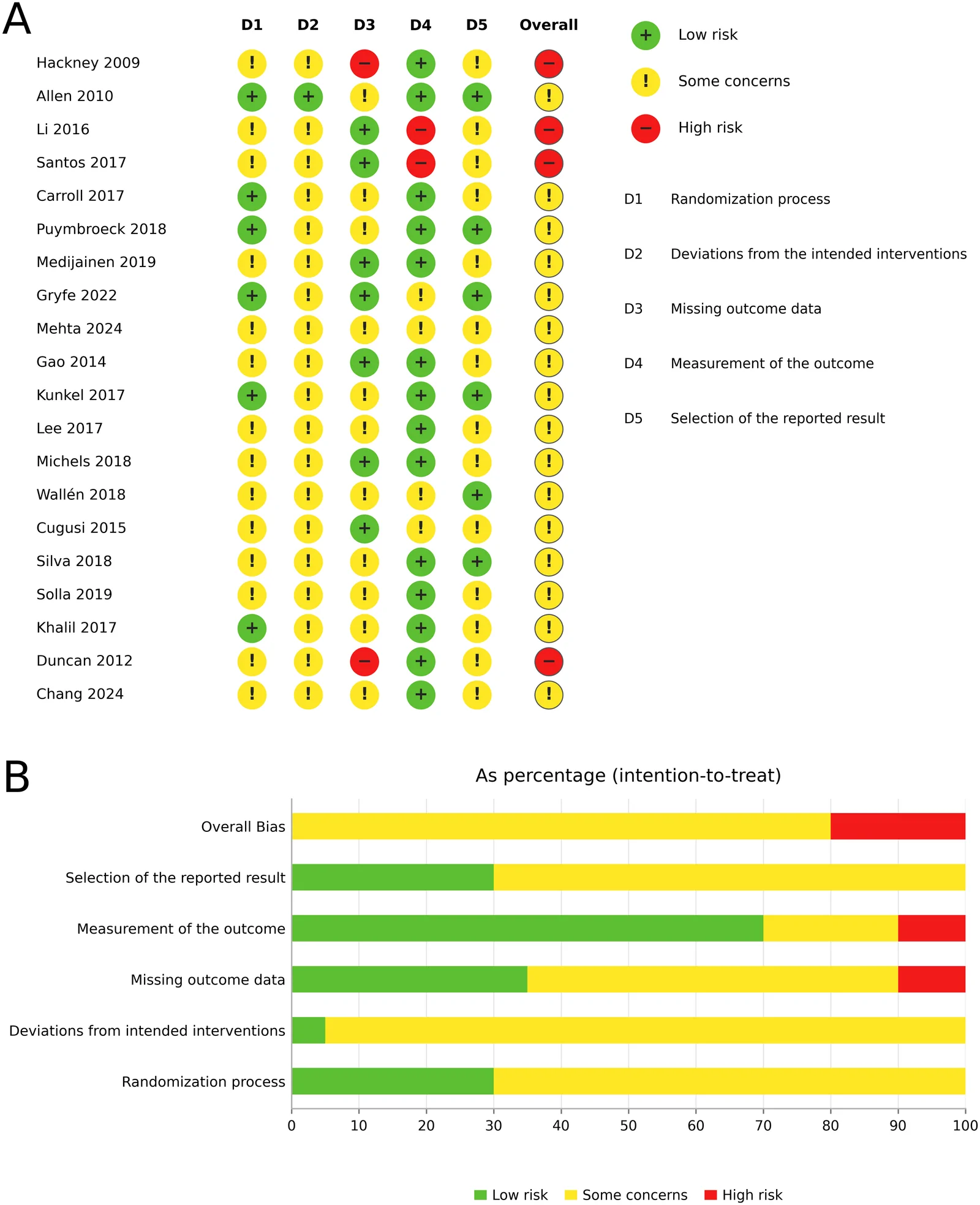
Risk of bias assessment using the Cochrane Risk of Bias 2 tool. (A) Traffic-light plot of study-level risk-of-bias judgments. (B) Summary plot of risk-of-bias judgments across RoB 2 domains.

Risk of bias in the included studies is summarized in Fig 2. Four of the 20 included RCTs had a high overall risk of bias, while the remaining 16 had some concerns; none was rated as low risk. For the randomization process, six studies were rated as low risk and 14 as having some concerns. For deviations from the intended interventions, most studies were judged as having some concerns, mainly because blinding of participants and intervention providers was difficult in exercise-intervention trials. For missing outcome data, seven studies were rated as low risk, 11 as having some concerns, and two as high risk. For measurement of the outcome, 14 studies were rated as low risk, four as having some concerns, and two as high risk. For selection of the reported result, six studies were rated as low risk and 14 as having some concerns. Overall, the main sources of bias were deviations from intended interventions, incomplete reporting of randomization procedures, missing outcome data, and outcome measurement.

### Characteristics of included studies

Twenty RCTs published between 2009 and 2024 were included, comprising 714 patients with PD, of whom 372 were assigned to exercise intervention groups and 342 to control groups [18–37]. The exercise interventions included dance-based training, Tai Chi, Qigong, yoga, treadmill training, balance/gait training, resistance training, aquatic exercise, walking training, and multimodal exercise. The outcome measures used in the meta-analysis included FOGQ, UPDRS-III, BBS, and Mini-BESTest, as shown in Table 2.

**Table 2:** Basic characteristics of the included studies.

Included studies	n		Sex (male/female), n		Age, years		Diagnostic criteria	Disease duration, years		Exercise modality		Intervention parameters	Outcome measures
	T	C	T	C	T	C		T	C	T	C		
Hackney 2009	14	17	11/3	12/5	68.2 ± 5.24	66.5 ± 11.54	Hoehn-Yahr 1–3	6.9 ± 4.86	5.9 ± 4.12	Argentine tango	Usual care	60 min per session, 2 times/week, for 13 weeks	①②③
Allen 2010	24	24	13/11	13/11	66 ± 10	68 ± 7	Clinical diagnosis	7 ± 6	9 ± 6	Lower-limb resistance training and balance training	Usual care	40–60 min per session, 3 times/week, for 24 weeks	①
Li 2016	14	14	7/7	8/6	67.6 ± 5.6	65.6 ± 6.1	Clinical diagnosis	7.5 ± 4.2	7.3 ± 3.7	treadmill training	Usual care	60 min per session, 5 times/week, for 4 weeks	①②
Santos 2017	10	10	5/5	5/5	73.09 ± 9.81	78.09 ± 5.24	Hoehn-Yahr 1–3	10.72 ± 4.14	10.90 ± 3.23	Balance training	Usual care	23 min per session, 2 times/week, for 6 weeks	①
Carroll 2017	10	8	7/3	5/3	69.67 ± 2.96	72.67 ± 7.41	Clinical diagnosis	7.5 ± 6.67	9.42 ± 6.85	Aquatic exercise	Usual care	45 min per session, 2 times/week, for 6 weeks	①②
Puymbroeck 2018	15	12	10/5	7/5	65.53 ± 6.09	70.50 ± 4.44	Hoehn-Yahr 1.5–3	NR	NR	Yoga	Usual care	60 min per session, 2 times/week, for 8 weeks	①②④
Medijainen 2019	12	12	5/7	5/7	71.1 ± 4.2	69.9 ± 5.1	Hoehn-Yahr	8.0 ± 6.9	7.7 ± 5.4	Combined training	Usual care	60 min per session, 2 times/week, for 8 weeks	①
Gryfe 2022	13	12	NR	NR	70.7 ± 7.3	69.3 ± 8.0	Hoehn-Yahr 1–4	NR	NR	Combined training	Usual care	60 min per session, 2 times/week, for 8 weeks	①
Mehta 2024	17	15	NR	NR	59.45 ± 9.37	62.13 ± 8.21	Hoehn-Yahr 1–2.5	3.26 ± 0.97	3.07 ± 0.94	Garba dance (India)	Usual care	60 min per session, 5 times/week, for 12 weeks	①④
Gao 2014	37	39	23/14	27/12	69.54 ± 7.32	68.28 ± 8.53	Clinical diagnosis	9.15 ± 8.58	8.37 ± 8.24	24-form Yang-style Tai Chi	Usual care	60 min per session, 3 times/week, for 12 weeks	②③
Kunkel 2017	31	31	NR	NR	71.3 ± 7.7	69.7 ± 6.0	Hoehn-Yahr 1–3	4.7 ± 3.5	7.0 ± 4.9	Partnered ballroom dance	Usual care	60 min per session, 2 times/week, for 10 weeks	③
Lee 2017	25	16	10/15	7/9	65.8 ± 7.2	65.7 ± 6.4	Hoehn-Yahr 1–3	4.5 ± 3.3	4.4 ± 3.0	Qigong dance	Usual care	60 min per session, 2 times/week, for 8 weeks	②③
Michels 2018	9	4	NR	NR	66.44 ± 8.7	75.50 ± 8.7	Clinical diagnosis	NR	NR	Dance therapy	Usual care	60 min per session, 1 time/week, for 10 weeks	②③
Wallén 2018	51	49	32/19	25/24	73.1 ± 5.8	73.0 ± 5.5	Clinical diagnosis	5.9 ± 5.1	5.6 ± 4.8	Highly challenging balance training	Usual care	60 min per session, 3 times/week, for 10 weeks	④
Cugusi 2015	10	10	8/2	8/2	68.1 ± 8.7	66.6 ± 7.3	Hoehn-Yahr 1–3	7 ± 2	7 ± 4	Walking training	Usual care	60 min per session, 2 times/week, for 12 weeks	②③
Silva 2018	14	11	6/8	5/6	63.12 ± 13.61	64.23 ± 3.45	Hoehn-Yahr 1–4	NR	NR	Aquatic exercise	Usual care	50 min per session, 2 times/week, for 10 weeks	③
Solla 2019	10	10	6/4	7/3	67.8 ± 5.9	67.1 ± 6.3	Hoehn-Yahr 1–3	4.4 ± 4.5	5.0 ± 2.9	Folk dance	Usual care	90 min per session, 2 times/week, for 12 weeks	②③
Khalil 2017	14	9	NR	NR	58.4 ± 13.5	60.7 ± 15.4	Hoehn-Yahr 1–4	8.0 ± 6.4	7.5 ± 4.0	Combined training	Usual care	45 min per session, 4 times/week, for 8 weeks	②④
Duncan 2012	26	26	15/11	15/11	69.3 ± 9.69	69.0 ± 7.65	Hoehn-Yahr 1–4	5.8 ± 5.61	7.0 ± 5.10	Argentine tango	Usual care	60 min per session, 2 times/week, for 52 weeks	②
Chang 2024a / Chang 2024b	16	13	7/9	6/7	66.31 ± 6.54	63.15 ± 7.95	Hoehn-Yahr 1–2	6.75 ± 5.49	6.77 ± 6.84	Aerobic exercise; Tai Chi Chuan	Usual care	60 min per session, 2 times/week, for 12 weeks	②

Note: T, intervention group; C, control group; (1) FOGQ; (2) UPDRS-III; (3) BBS; (4) Mini-BESTest; NR, not reported. Two included studies required arm-level handling. For Li 2016, only the exercise-only treadmill-training arm was retained, whereas the treadmill training plus music co-intervention arm was excluded from the quantitative synthesis. The shared comparator group was used only once with the retained exercise-only arm and was not duplicated. For Chang 2024, two eligible exercise-only arms, aerobic exercise and Tai Chi Chuan, shared the same control group. Both exercise arms were included as separate comparisons in the UPDRS-III meta-analysis, and the shared control group was handled to avoid double counting. Medication state at outcome assessment and medication-regimen stability during the intervention were extracted when reported; otherwise, they were coded as NR.

### Meta-analysis

#### FOGQ.

**Fig 3:**
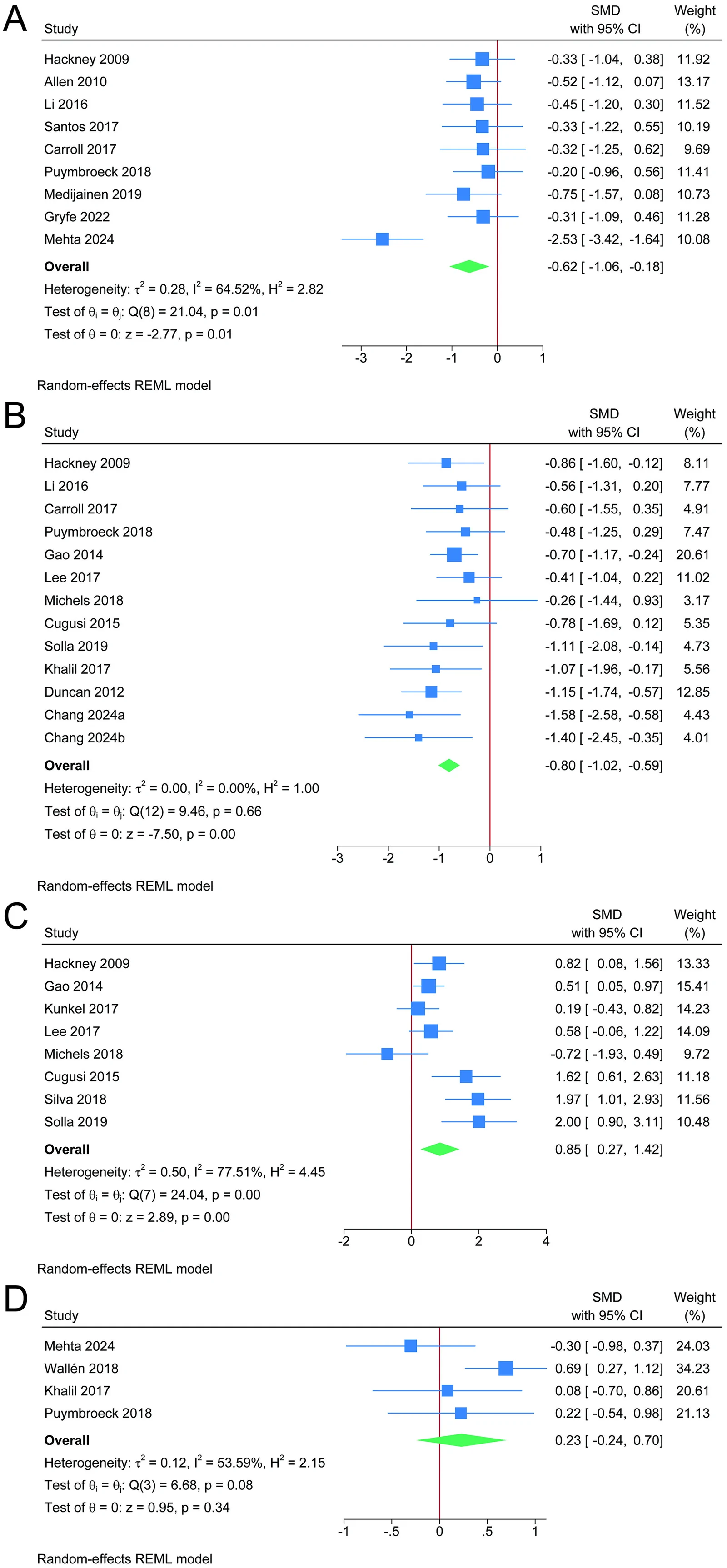
Meta-analysis of the effects of exercise interventions on gait-related, motor-function, and balance outcomes in patients with PD. Chang 2024a and Chang 2024b refer to two eligible exercise-intervention arms from the same multi-arm trial reported by Chang et al. 2024. (A) FOGQ; (B) UPDRS-III; (C) BBS; (D) Mini-BESTest.

Nine studies reported FOGQ outcomes in patients with PD [18–26]. Results were pooled using a prespecified random-effects REML model. Compared with control, exercise interventions were associated with a significantly lower FOGQ score [SMD=−0.62, 95% CI (−1.06, −0.18), P = 0.01]. Heterogeneity was moderate (I² = 64.52%, τ² = 0.28,P = 0.01). Fig 3A.

In the leave-one-out analysis for FOGQ, sequential omission of each study yielded pooled estimates ranging from −0.67 to −0.41, and all remained statistically significant (all P ≤ 0.017). Further details are provided in Appendix Fig 1.

#### UPDRS-III.

Thirteen comparisons reported UPDRS-III outcomes in patients with PD [18,20,22,23,27,29,30,32,34–37]. Results were pooled using a prespecified random-effects REML model. Compared with control, exercise interventions were associated with a significantly lower UPDRS-III score [SMD=−0.80, 95% CI (−1.02, −0.59), P < 0.001]. Heterogeneity was low (I² = 0.00%, τ² = 0.00,P = 0.66). Fig 3B.

In the leave-one-out analysis for UPDRS-III, sequential omission of each comparison yielded pooled estimates ranging from −0.85 to −0.75, and all remained statistically significant (all P < 0.001). Further details are provided in Appendix Fig 2.

#### BBS.

Eight studies reported BBS outcomes in patients with PD [18,27–30,32–34]. Results were pooled using a prespecified random-effects REML model. Compared with control, exercise interventions were associated with a significantly higher BBS score [SMD = 0.85, 95% CI (0.27, 1.42), P = 0.004]. Heterogeneity was high (I² = 77.51%, τ² = 0.50,P = 0.00). Fig 3C.

In the leave-one-out analysis for BBS, sequential omission of each study yielded pooled estimates ranging from 0.69 to 0.99, and all remained statistically significant (all P ≤ 0.017), indicating that the overall result was not driven by any single study. Further details are provided in Appendix Fig 3.

#### Mini-BESTest.

Four studies reported Mini-BESTest outcomes in patients with PD [23,26,31,35]. Results were pooled using a prespecified random-effects REML model. Exercise interventions showed a trend towards improved Mini-BESTest scores but did not reach statistical significance [SMD = 0.23, 95% CI (−0.24, 0.70), P = 0.34]. Heterogeneity was moderate (I^2^ = 53.59%, τ² = 0.12,P = 0.08). Fig 3D.

In the leave-one-out analysis for Mini-BESTest, sequential omission of each study yielded pooled estimates ranging from −0.03 to 0.44. However, statistical significance was not consistently maintained across iterations: only one omitted-study model remained significant (P = 0.039), whereas the others were non-significant (P = 0.414–0.903). Further details are provided in Appendix Fig 4.

**Fig 4:**
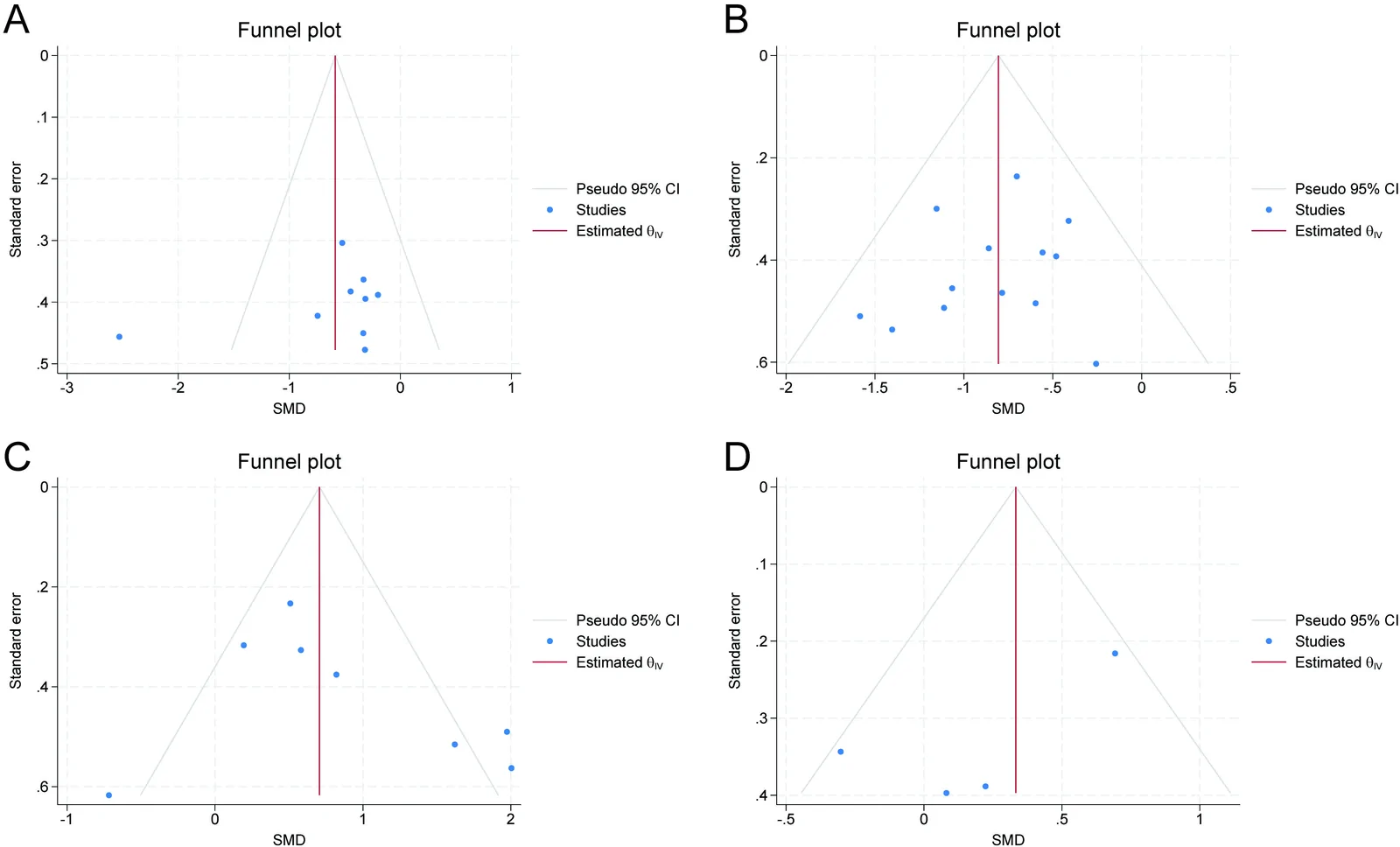
Funnel plots for assessment of publication bias in exercise intervention effects. (A) FOGQ; (B) UPDRS-III; (C) BBS; (D) Mini-BESTest.

### Subgroup analyses

Prespecified subgroup analyses were conducted for FOGQ, UPDRS-III, and BBS according to age, disease duration, intervention duration, training frequency, single-session training time, and exercise modality. These analyses were exploratory and were intended only to examine potential sources of heterogeneity. No formal tests for between-subgroup differences or interaction effects were performed, and several subgroup estimates were based on a small number of studies. Differences in nominal statistical significance across subgroups do not constitute evidence of a between-subgroup difference. The subgroup findings are presented in Tables 3, 4, 5 and should be regarded as hypothesis-generating only. Subgroup analysis by Hoehn–Yahr stage was not performed because the original trials generally did not report outcome data separately by Hoehn–Yahr stage. Most studies included mixed-stage samples and reported only a stage range or group-level disease-severity summary. Conducting a study-level subgroup analysis using only the reported stage range would have been ecologically vulnerable and could have produced misleading inferences. Therefore, Hoehn–Yahr stage was summarized descriptively rather than used as a moderator in the meta-analysis.

**Table 3:** Subgroup analysis results for FOGQ.

Subgroup		Number of included studies, n	Test for heterogeneity		Meta-analysis		
			P value	I^2^ (%)	Z value	SMD (95%CI)	P value
Age	59–68 years	4	<0.001	86.76	−1.73	−0.90 (−1.92 to 0.12)	0.08
	69–78 years	5	0.94	0.00	−2.19	−0.41 (−0.77 to −0.04)	0.03
Intervention duration	≤10 weeks	6	0.96	0.00	−2.31	−0.39 (−0.72 to −0.06)	0.02
	>10 weeks	3	<0.001	90.32	−1.60	−1.10 (−2.44 to 0.25)	0.11
Single-session intervention time	≥60 min	7	<0.001	74.12	−2.46	−0.70 (−1.26 to −0.14)	0.01
	<60 min	2	0.98	0.00	−1.00	−0.33 (−0.97 to 0.32)	0.32
Intervention frequency	≤2 times/week	6	0.96	0.00	−2.20	−0.37 (−0.70 to −0.04)	0.03
	>2 times/week	3	<0.001	89.32	−1.71	−1.14 (−2.44 to 0.17)	0.09
Exercise modality	Single-modality exercise	4	0.98	0.00	−1.56	−0.33 (−0.74 to 0.08)	0.12
	Combined/multimodal training	5	<0.001	81.49	−2.16	−0.86 (−1.64 to −0.08)	0.03

Note: The disease-duration subgroup analysis was not performed for the ≤ 6 years category because only one study was available. Therefore, no pooled effect estimate or heterogeneity statistic was calculated for this subgroup.

**Table 4:** Subgroup analysis results for UPDRS-III.

Subgroup		Number of included studies, n	Test for heterogeneity		Meta-analysis		
			P value	I² (%)	Z value	SMD (95%CI)	P value
Age	59–68 years	9	0.51	0.00	−5.27	−0.76 (−1.04 to −0.47)	<0.001
	69–78 years	3	0.43	0.00	−4.85	−0.84 (−1.18 to −0.50)	<0.001
Disease duration	≤6 years	3	0.21	39.42	−3.25	−0.87 (−1.39 to −0.34)	0.001
	>6 years	8	0.71	0.00	−6.15	−0.85 (−1.12 to −0.58)	<0.001
Intervention duration	≤10 weeks	6	0.88	0.00	−3.26	−0.55 (−0.89 to −0.22)	0.001
	>10 weeks	7	0.66	0.00	−7.01	−0.97 (−1.24 to −0.70)	<0.001
Single-session intervention time	≥60 min	11	0.54	0.00	−7.05	−0.80 (−1.02 to −0.58)	<0.001
	<60 min	2	0.48	0.00	−2.55	−0.85 (−1.50 to −0.20)	0.01
Intervention frequency	≤2 times/week	10	0.49	1.48	−6.33	−0.84 (−1.11 to −0.58)	<0.001
	>2 times/week	3	0.68	0.00	−3.95	−0.73 (−1.09 to −0.37)	<0.001
Exercise modality	Single-modality exercise	9	0.57	0.00	−5.77	−0.74 (−0.99 to −0.49)	<0.001
	Combined/multimodal training	4	0.59	0.00	−4.88	−0.96 (−1.35 to −0.57)	<0.001

**Table 5:** Subgroup analysis results for BBS.

Subgroup		Number of included studies, n	Test for heterogeneity		Meta-analysis		
			P value	I^2^ (%)	Z value	SMD (95%CI)	P value
Age	59–68 years	4	<0.001	84.84	1.57	0.97 (−0.24 to 2.19)	0.12
	69–78 years	2	0.42	0.00	2.12	0.40 (0.03 to 0.77)	0.03
Disease duration	≤6 years	3	0.02	80.36	1.66	0.83 (−0.15 to 1.82)	0.10
	>6 years	3	0.14	48.71	2.93	0.85 (0.28 to 1.41)	<0.001
Intervention duration	≤10 weeks	4	<0.001	83.95	1.03	0.53 (−0.48 to 1.54)	0.30
	>10 weeks	4	0.04	66.12	3.24	1.11 (0.44 to 1.78)	<0.001
Exercise modality	Single-modality exercise	5	0.01	73.42	3.57	1.23 (0.55 to 1.90)	<0.001
	Combined/multimodal training	3	0.09	59.97	0.57	0.22 (−0.53 to 0.96)	0.57

Note: For BBS, subgroup analyses were not performed for the < 60 min/session category and the > 2 times/week category because each subgroup included only one study. Therefore, pooled effect estimates and heterogeneity statistics were not calculated for these subgroups.

### Publication bias

Funnel plots for FOGQ, UPDRS-III, BBS, and Mini-BESTest are shown in Fig 4. For UPDRS-III, which included at least 10 comparisons, funnel plot inspection and Egger’s regression test were performed as a formal assessment.

Egger’s regression test was not statistically significant for UPDRS-III (P = 0.5022), suggesting no evidence of significant small-study effects or publication bias. For FOGQ, BBS, and Mini-BESTest, because each outcome included fewer than 10 studies, funnel plot inspection and Egger’s regression tests were performed only as exploratory and underpowered assessments. The exploratory Egger’s test P values were 0.3465 for FOGQ, 0.4665 for BBS, and 0.0927 for Mini-BESTest. These results should be interpreted cautiously and cannot exclude publication bias or small-study effects.

### Certainty of evidence assessment

The GRADE assessment showed that the certainty of evidence was moderate for UPDRS-III and low for FOGQ, BBS, and Mini-BESTest. The downgrading was mainly due to risk of bias, inconsistency, and imprecision. The detailed GRADE ratings are shown in Table 6.

**Table 6:** GRADE certainty of evidence.

Outcome measure	Number of included studies/comparisons, n	Certainty of evidence					Overall certainty
		Risk of bias	Inconsistency	Indirectness	Imprecision	Publication bias	
FOGQ	9	serious^a^	serious^b^	Not serious^c^	Not serious	Not seriousᵉ	Low
UPDRS-III	13	serious^a^	Not serious^b^	Not serious^c^	Not serious	Not seriousᵉ	Moderate
BBS	8	serious^a^	serious^b^	Not serious^c^	Not serious	Not seriousᵉ	Low
Mini-BESTest	4	serious^a^	Not serious^b^	Not serious^c^	seriousᵈ	Not seriousᵉ	Low

Notes: ^a^ Downgraded one level for risk of bias because the included RCTs had methodological limitations. Four studies were judged as having a high overall risk of bias, and the remaining studies were rated as having some concerns; the main sources of bias included deviations from intended interventions, incomplete reporting of randomization procedures, missing outcome data, and outcome measurement. ^b^ FOGQ and BBS were downgraded one level for inconsistency because heterogeneity was moderate to high and the magnitude of effects varied across studies, with sources of heterogeneity not fully explained. UPDRS-III was not downgraded for inconsistency because heterogeneity was low. Mini-BESTest was not additionally downgraded for inconsistency because the moderate heterogeneity estimate was based on only four studies and was considered together with imprecision. ^c^ Indirectness was not downgraded because the populations, interventions, comparators, and outcomes were directly relevant to the review question. ^d^ Mini-BESTest was downgraded one level for imprecision because only four studies were included, the 95% confidence interval crossed the line of no effect, and the leave-one-out sensitivity analysis showed unstable statistical significance. FOGQ, UPDRS-III, and BBS were not downgraded for imprecision because their pooled estimates excluded the line of no effect and their sensitivity analyses did not indicate that the overall results were driven by a single study. ^e^ Publication bias was not downgraded. Formal assessment was performed for UPDRS-III, and Egger’s test was not statistically significant. For FOGQ, BBS, and Mini-BESTest, fewer than 10 studies were included, so publication-bias assessment was exploratory and underpowered; therefore, publication bias could not be excluded, but there was no clear evidence warranting downgrading.

## Discussion

This systematic review and meta-analysis included 20 RCTs involving 714 patients with PD. Pooled estimates favored exercise interventions for FOGQ, UPDRS-III, and BBS compared with usual care, usual rehabilitation, or no structured exercise, whereas the Mini-BESTest effect was not statistically significant. Evidence certainty was moderate for UPDRS-III and low for FOGQ, BBS, and Mini-BESTest, warranting cautious interpretation.

Most included trials had small sample sizes, which may have reduced the stability and precision of the SMD estimates. This limitation was particularly relevant to the Mini-BESTest, for which only four studies were available and the leave-one-out analysis indicated limited robustness. The outcome measures also differed in their clinical scope: FOGQ reflects gait-related symptoms, BBS and Mini-BESTest assess balance performance, and UPDRS-III primarily evaluates overall motor function and has limited specificity for lower-limb function [3,12]. Accordingly, the findings are most informative regarding gait-related symptoms, overall motor function, and balance performance, while their implications for lower-limb-specific function remain indirect [3,5].

The overall pattern of findings was broadly consistent with previous evidence. Previous network meta-analyses have suggested that structured exercise can improve motor symptoms and balance in patients with PD, although evidence supporting the superiority of specific exercise modalities remains limited [38–40]. However, reviews focusing on dance-based interventions have reported non-significant effects and low-certainty evidence indicating continued uncertainty across intervention modalities [41]. The inconclusive Mini-BESTest result may reflect the small number of contributing studies, wide confidence intervals, and differences in the balance domains and measurement properties of the Mini-BESTest and BBS [12]. Although a scale-specific minimal clinically important difference has been proposed for the Mini-BESTest [42], the use of SMDs and the limited number of contributing studies precluded direct assessment of whether the pooled effect was clinically meaningful.

The exploratory subgroup analyses showed some variation in pooled estimates by age, disease duration, intervention duration, training frequency, single-session duration, and exercise modality. Interpretation of the findings for age and disease duration is particularly complex because age at disease onset and age-related postural instability may influence the clinical profile of PD independently of disease duration [43–46]. These findings should be interpreted cautiously because formal interaction tests were not performed and several subgroup estimates were based on a small number of studies. Differences in nominal statistical significance alone do not constitute evidence of between-subgroup differences [15]; therefore, the current analyses do not establish differential effects according to exercise modality, dose parameters, age, or disease duration. The observed patterns may nevertheless help identify potential sources of heterogeneity and inform the design of future adequately powered trials and individual participant data meta-analyses. Future trials should directly compare exercise modalities and dose parameters, use standardized outcome measures, and provide detailed information on intervention intensity, adherence, supervision, and control conditions, while future meta-analyses should prespecify interaction tests when evaluating potential effect modifiers [47–49].

Exercise interventions may improve freezing-of-gait symptoms, overall motor function, and balance performance as assessed by the BBS, whereas evidence based on the Mini-BESTest remains inconclusive. Potential explanations for these findings involve two complementary mechanisms: peripheral neuromuscular adaptation and central reorganization of motor-control networks. First, repeated exercise may induce structural and functional adaptations in the lower-limb and trunk musculature and the postural-control system, thereby improving muscle strength, fatigue resistance, motor-unit recruitment, intermuscular coordination, and the efficiency of postural and gait control [50–52]. Second, gait and balance depend on integrated regulation within cortico–basal ganglia–cerebellar and brainstem circuits, which are disrupted in PD and contribute to impaired automatic motor control [4,53,54]. Through activity- and experience-dependent neuroplasticity, exercise training may modulate cortical excitability and inhibitory control, enhance sensorimotor integration, and promote adaptive reorganization of cortico-striatal and related motor networks, thereby supporting improvements in gait execution and postural stability [55–57]. Training-induced structural brain plasticity may also contribute to improvements in balance performance [58]. However, these molecular, cellular, and neural pathways were not directly evaluated in the included RCTs and should therefore be considered biologically plausible explanatory hypotheses requiring confirmation in future mechanistic studies.

This study has several limitations. First, medication status and dopaminergic treatment were incompletely reported across the included trials. This prevented medication-stratified analyses and may have contributed to between-study heterogeneity. Second, participants ranged from Hoehn–Yahr stage 1 to stage 4, but stage-specific outcome data were generally unavailable. Among trials reporting disease duration, the mean disease duration was approximately 6.6 years. Therefore, the findings may not be directly applicable to newly diagnosed patients, those with substantially longer disease duration, or patients at markedly different disease stages. Third, the included outcomes mainly assessed gait-related symptoms, balance, and overall motor function. They did not specifically assess isolated lower-limb function. In addition, the strict eligibility criteria may limit generalizability beyond structured exercise-only interventions. Finally, the evidence base was limited by small trial samples, the small number of studies reporting Mini-BESTest, the absence of trials with a low overall risk of bias, and residual clinical and methodological heterogeneity. Future studies should use rigorous trial methods and improve the reporting of medication status, disease stage, and intervention characteristics.

## Conclusion

Current evidence suggests that exercise interventions may improve freezing-of-gait symptoms, overall motor function, and some aspects of balance performance in patients with PD, although evidence for Mini-BESTest outcomes remains inconclusive. Evidence certainty was moderate for UPDRS-III and low for FOGQ, BBS, and Mini-BESTest. Exploratory subgroup analyses did not establish differential effects according to exercise modality, dose parameters, age, or disease duration. Further adequately powered randomized trials are needed to clarify the comparative effects of different exercise modalities and doses.

## Generative AI statement

The authors declare that no generative artificial intelligence was used in the preparation of this manuscript.

## Publisher’s note

All views expressed in this article are solely those of the authors and do not necessarily represent those of their affiliated organisations, or those of the publisher, the editors, and the reviewers. Any product that may be evaluated in this article, or any claim that may be made by its manufacturer, is not guaranteed or endorsed by the publisher.

## Supporting information

S1 File Search strategies, reporting checklists, and sensitivity analyses. Complete database search strategies, the PRISMA 2020 and PRISMA-S checklists, and leave-one-out sensitivity analyses for FOGQ, UPDRS-III, BBS, and Mini-BESTest.
(DOC)

S2 File Extracted dataset used for the meta-analysis. Study-level aggregate data extracted from the included studies, including intervention characteristics, group sample sizes, and pre- and post-intervention means and standard deviations.
(XLSX)

## Abbreviations

PD: Parkinson’s disease
PICOS: Population, Intervention, Comparator, Outcomes, and Study design
PRISMA: Preferred Reporting Items for Systematic Reviews and Meta-Analyses
PROSPERO: International Prospective Register of Systematic Reviews
RCT: randomized controlled trial
SMD: standardized mean difference
UPDRS-III: Unified Parkinson’s Disease Rating Scale Part III
Mini-BESTest: Mini-Balance Evaluation Systems Test
GRADE: Grading of Recommendations Assessment, Development and Evaluation
FOGQ: Freezing of Gait Questionnaire
CENTRAL: Cochrane Central Register of Controlled Trials
BBS: Berg Balance Scale
CI: Confidence interval
REML: Restricted maximum likelihood
RoB 2: Cochrane Risk of Bias 2.
