# Supplementary material for: Effects of exercise interventions on gait, motor function, and balance in patients with Parkinson’s disease: A meta-analysis with clinical implications
Source: PLoS One. 2026 Sep 25;21(9):e0359274. doi: 10.1371/journal.pone.0359274 (PMC13614582; doi:10.1371/journal.pone.0359274)
Supplement: Supplementary file 1 [file pone.0359274.s001.DOC]

**Appendix 1. Complete search strategies**

**The literature search was conducted in PubMed, Embase, Web of Science, Scopus, and Cochrane Library/CENTRAL from database inception to January 10, 2026. No language restriction was applied. Searches were limited to human studies and randomized or controlled clinical trial designs where such filters were available. Reference lists of included studies and relevant reviews were manually screened, and citation searching was conducted to identify additional eligible randomized controlled trials.**

| **January 10, 2026** | **PubMed (607)** |
| --- | --- |
| #1 | "Parkinson Disease"[Mesh] OR "Parkinsonian Disorders"[Mesh] OR parkinson*[tiab] OR parkinsonian[tiab] OR parkinsonism[tiab] OR "paralysis agitans"[tiab] |
| #2 | "Exercise"[Mesh] OR "Exercise Therapy"[Mesh] OR "Physical Therapy Modalities"[Mesh] OR "Rehabilitation"[Mesh] OR "Resistance Training"[Mesh] OR "Walking"[Mesh] OR "Dance Therapy"[Mesh] OR exercise*[tiab] OR "exercise intervention*"[tiab] OR "exercise therap*"[tiab] OR "physical activit*"[tiab] OR training[tiab] OR "physical training"[tiab] OR rehabilitation[tiab] OR physiotherapy[tiab] OR "physical therap*"[tiab] OR aerobic[tiab] OR resistance[tiab] OR strengthening[tiab] OR "strength training"[tiab] OR balance[tiab] OR "balance training"[tiab] OR gait[tiab] OR "gait training"[tiab] OR treadmill[tiab] OR walking[tiab] OR dance[tiab] OR tango[tiab] OR "tai chi"[tiab] OR taiji[tiab] OR yoga[tiab] OR qigong[tiab] OR aquatic[tiab] |
| #3 | randomized controlled trial[pt] OR controlled clinical trial[pt] OR randomi*ed[tiab] OR randomly[tiab] OR randomization[tiab] OR randomisation[tiab] OR placebo[tiab] OR trial[ti] NOT (animals[mh] NOT humans[mh]) |
| #4 | #1 AND #2 AND #3 |

| **January 10, 2026** | **Embase (2257)** |
| --- | --- |
| #1 | ('parkinson disease'/exp OR 'parkinsonian disorder'/exp OR parkinson*:ti,ab,kw OR parkinsonian*:ti,ab,kw OR parkinsonism:ti,ab,kw OR 'paralysis agitans':ti,ab,kw) |
| #2 | ('exercise'/exp OR 'exercise therapy'/exp OR 'physical activity'/exp OR 'physiotherapy'/exp OR 'rehabilitation'/exp OR 'resistance training'/exp OR 'aerobic exercise'/exp OR 'walking'/exp OR 'dance therapy'/exp OR exercise*:ti,ab,kw OR 'exercise intervention*':ti,ab,kw OR 'exercise therap*':ti,ab,kw OR 'physical activit*':ti,ab,kw OR training:ti,ab,kw OR 'physical training':ti,ab,kw OR rehabilitation:ti,ab,kw OR physiotherapy:ti,ab,kw OR 'physical therap*':ti,ab,kw OR aerobic:ti,ab,kw OR resistance:ti,ab,kw OR strengthening:ti,ab,kw OR 'strength training':ti,ab,kw OR balance:ti,ab,kw OR 'balance training':ti,ab,kw OR gait:ti,ab,kw OR 'gait training':ti,ab,kw OR treadmill:ti,ab,kw OR walking:ti,ab,kw OR dance:ti,ab,kw OR tango:ti,ab,kw OR 'tai chi':ti,ab,kw OR taiji:ti,ab,kw OR yoga:ti,ab,kw OR qigong:ti,ab,kw OR aquatic:ti,ab,kw) |
| #3 | ('randomized controlled trial'/exp OR randomization/exp OR 'controlled clinical trial'/exp OR randomi*ed:ti,ab,kw OR randomly:ti,ab,kw OR randomization:ti,ab,kw OR randomisation:ti,ab,kw OR placebo*:ti,ab,kw OR 'clinical trial*':ti,ab,kw OR 'controlled trial*':ti,ab,kw) NOT ('animal'/exp NOT 'human'/exp) |
| #4 | #1 AND #2 AND #3 |

| **January 10, 2026** | **Web of Science (2184)** |
| --- | --- |
| #1 | TS=("Parkinson disease" OR "Parkinson's disease" OR parkinson* OR "parkinsonian disorder*" OR parkinsonism OR "paralysis agitans") |
| #2 | TS=(exercise* OR "exercise intervention*" OR "exercise therap*" OR "physical activit*" OR training OR "physical training" OR rehabilitation OR physiotherapy OR "physical therap*" OR aerobic OR resistance OR strengthening OR "strength training" OR balance OR "balance training" OR gait OR "gait training" OR treadmill OR walking OR dance OR tango OR "tai chi" OR taiji OR yoga OR qigong OR aquatic) |
| #3 | TS=(randomi*ed OR randomly OR randomization OR randomisation OR "random allocation" OR "randomized controlled trial*" OR "controlled clinical trial*" OR "clinical trial*" OR "controlled trial*" OR placebo*) |
| #4 | #1 AND #2 AND #3 |

| **January 10, 2026** | **Scopus (2554)** |
| --- | --- |
| #1 | TITLE-ABS-KEY("Parkinson disease" OR "Parkinson's disease" OR parkinson* OR "parkinsonian disorder*" OR parkinsonism OR "paralysis agitans") |
| #2 | TITLE-ABS-KEY(exercise* OR "exercise intervention*" OR "exercise therap*" OR "physical activit*" OR training OR "physical training" OR rehabilitation OR physiotherapy OR "physical therap*" OR aerobic OR resistance OR strengthening OR "strength training" OR balance OR "balance training" OR gait OR "gait training" OR treadmill OR walking OR dance OR tango OR "tai chi" OR taiji OR yoga OR qigong OR aquatic) |
| #3 | TITLE-ABS-KEY(randomi*ed OR randomly OR randomization OR randomisation OR "random allocation" OR "randomized controlled trial*" OR "controlled clinical trial*" OR "clinical trial*" OR "controlled trial*" OR placebo*) |
| #4 | #1 AND #2 AND #3 |

| **January 10, 2026** | **Cochrane Library/CENTRAL (1696)** |
| --- | --- |
| #1 | (parkinson* OR "Parkinson disease" OR "Parkinson's disease" OR "parkinsonian disorder*" OR parkinsonism OR "paralysis agitans"):ti,ab,kw |
| #2 | (exercise* OR "exercise intervention*" OR "exercise therap*" OR "physical activit*" OR training OR "physical training" OR rehabilitation OR physiotherapy OR "physical therap*" OR aerobic OR resistance OR strengthening OR "strength training" OR balance OR "balance training" OR gait OR "gait training" OR treadmill OR walking OR dance OR tango OR "tai chi" OR taiji OR yoga OR qigong OR aquatic):ti,ab,kw |
| #3 | (randomi*ed OR randomly OR randomization OR randomisation OR placebo* OR "randomized controlled trial*" OR "clinical trial*" OR "controlled trial*"):ti,ab,kw |
| #4 | #1 AND #2 AND #3 |

| **Section and Topic** | **Item #** | **Checklist item** | **Location where item is reported** |
| --- | --- | --- | --- |
| **TITLE** | | |  |
| Title | 1 | Identify the report as a systematic review. | Title page |
| **ABSTRACT** | | |  |
| Abstract | 2 | See the PRISMA 2020 for Abstracts checklist. | Abstract, p. 2 |
| **INTRODUCTION** | | |  |
| Rationale | 3 | Describe the rationale for the review in the context of existing knowledge. | Introduction, pp. 2–3 |
| Objectives | 4 | Provide an explicit statement of the objective(s) or question(s) the review addresses. | Introduction, p. 3 |
| **METHODS** | | |  |
| Eligibility criteria | 5 | Specify the inclusion and exclusion criteria for the review and how studies were grouped for the syntheses. | Methods: Eligibility criteria, pp. 3–4 |
| Information sources | 6 | Specify all databases, registers, websites, organisations, reference lists and other sources searched or consulted to identify studies. Specify the date when each source was last searched or consulted. | Methods: Literature search strategy, p. 3; Appendix 1 |
| Search strategy | 7 | Present the full search strategies for all databases, registers and websites, including any filters and limits used. | Methods: Literature search strategy, p. 3; Appendix 1 |
| Selection process | 8 | Specify the methods used to decide whether a study met the inclusion criteria of the review, including how many reviewers screened each record and each report retrieved, whether they worked independently, and if applicable, details of automation tools used in the process. | Methods: Study selection and data extraction, p. 4; Figure 1 |
| Data collection process | 9 | Specify the methods used to collect data from reports, including how many reviewers collected data from each report, whether they worked independently, any processes for obtaining or confirming data from study investigators, and if applicable, details of automation tools used in the process. | Methods: Study selection and data extraction, p. 4 |
| Data items | 10a | List and define all outcomes for which data were sought. Specify whether all results that were compatible with each outcome domain in each study were sought (e.g. for all measures, time points, analyses), and if not, the methods used to decide which results to collect. | Methods: Eligibility criteria; Study selection and data extraction, pp. 3–4 |
|  | 10b | List and define all other variables for which data were sought (e.g. participant and intervention characteristics, funding sources). Describe any assumptions made about any missing or unclear information. | Methods: Study selection and data extraction, p. 4; Table 2; Supplementary File 2 |
| Study risk of bias assessment | 11 | Specify the methods used to assess risk of bias in the included studies, including details of the tool(s) used, how many reviewers assessed each study and whether they worked independently, and if applicable, details of automation tools used in the process. | Methods: Methodological quality assessment, p. 4; Figure 2 |
| Effect measures | 12 | Specify for each outcome the effect measure(s) (e.g. risk ratio, mean difference) used in the synthesis or presentation of results. | Methods: Statistical analysis, pp. 4–5 |
| Synthesis methods | 13a | Describe the processes used to decide which studies were eligible for each synthesis (e.g. tabulating the study intervention characteristics and comparing against the planned groups for each synthesis (item #5)). | Methods: Eligibility criteria; Statistical analysis, pp. 3–5 |
|  | 13b | Describe any methods required to prepare the data for presentation or synthesis, such as handling of missing summary statistics, or data conversions. | Methods: Eligibility criteria; Study selection and data extraction; Statistical analysis, pp. 3–5; Supplementary File 2 |
|  | 13c | Describe any methods used to tabulate or visually display results of individual studies and syntheses. | Results: Meta-analysis and subgroup analyses, pp. 5–7; Figures 3–4; Tables 3–6 |
|  | 13d | Describe any methods used to synthesize results and provide a rationale for the choice(s). If meta-analysis was performed, describe the model(s), method(s) to identify the presence and extent of statistical heterogeneity, and software package(s) used. | Methods: Statistical analysis, pp. 4–5 |
|  | 13e | Describe any methods used to explore possible causes of heterogeneity among study results (e.g. subgroup analysis, meta-regression). | Methods: Statistical analysis, pp. 4–5; Results: Subgroup Analyses, pp. 6–7; Tables 3–5 |
|  | 13f | Describe any sensitivity analyses conducted to assess robustness of the synthesized results. | Methods: Statistical analysis, pp. 4–5; Results: Meta-analysis, pp. 5–6; Appendix Figures 1–4 |
| Reporting bias assessment | 14 | Describe any methods used to assess risk of bias due to missing results in a synthesis (arising from reporting biases). | Methods: Statistical analysis, pp. 4–5 |
| Certainty assessment | 15 | Describe any methods used to assess certainty (or confidence) in the body of evidence for an outcome. | Methods: Certainty of evidence assessment, p. 5 |
| **RESULTS** | | |  |
| Study selection | 16a | Describe the results of the search and selection process, from the number of records identified in the search to the number of studies included in the review, ideally using a flow diagram. | Results: Study selection, p. 5; Figure 1 |
|  | 16b | Cite studies that might appear to meet the inclusion criteria, but which were excluded, and explain why they were excluded. | Figure 1; full-text exclusion reasons summarized in PRISMA flow diagram |
| Study characteristics | 17 | Cite each included study and present its characteristics. | Results: Characteristics of included studies, p. 5; Table 2 |
| Risk of bias in studies | 18 | Present assessments of risk of bias for each included study. | Results: Risk of bias in included studies, p. 5; Figure 2 |
| Results of individual studies | 19 | For all outcomes, present, for each study: (a) summary statistics for each group (where appropriate) and (b) an effect estimate and its precision (e.g. confidence/credible interval), ideally using structured tables or plots. | Results: Meta-analysis, pp. 5–6; Figure 3; Supplementary File 2 |
| Results of syntheses | 20a | For each synthesis, briefly summarise the characteristics and risk of bias among contributing studies. | Results: Meta-analysis, pp. 5–6; Figure 2; Figure 3; Table 6 |
|  | 20b | Present results of all statistical syntheses conducted. If meta-analysis was done, present for each the summary estimate and its precision (e.g. confidence/credible interval) and measures of statistical heterogeneity. If comparing groups, describe the direction of the effect. | Results: Meta-analysis, pp. 5–6; Figure 3; Tables 3–6 |
|  | 20c | Present results of all investigations of possible causes of heterogeneity among study results. | Results: Subgroup Analyses, pp. 6–7; Tables 3–5 |
|  | 20d | Present results of all sensitivity analyses conducted to assess the robustness of the synthesized results. | Results: Meta-analysis, pp. 5–6; Appendix Figures 1–4 |
| Reporting biases | 21 | Present assessments of risk of bias due to missing results (arising from reporting biases) for each synthesis assessed. | Results: Publication bias, p. 7; Figure 4 |
| Certainty of evidence | 22 | Present assessments of certainty (or confidence) in the body of evidence for each outcome assessed. | Results: Certainty of evidence assessment, p. 7; Table 6 |
| **DISCUSSION** | | |  |
| Discussion | 23a | Provide a general interpretation of the results in the context of other evidence. | Discussion, pp. 7–8 |
|  | 23b | Discuss any limitations of the evidence included in the review. | Discussion: Limitations, pp. 8–9 |
|  | 23c | Discuss any limitations of the review processes used. | Discussion: Limitations, pp. 8–9 |
|  | 23d | Discuss implications of the results for practice, policy, and future research. | Discussion and Conclusion, pp. 8–9 |
| **OTHER INFORMATION** | | |  |
| Registration and protocol | 24a | Provide registration information for the review, including register name and registration number, or state that the review was not registered. | Abstract, p. 2; Methods: Study framework, p. 3; PROSPERO CRD420261281718 |
|  | 24b | Indicate where the review protocol can be accessed, or state that a protocol was not prepared. | PROSPERO registry, CRD420261281718 |
|  | 24c | Describe and explain any amendments to information provided at registration or in the protocol. | Not applicable; no protocol amendments were made. |
| Support | 25 | Describe sources of financial or non-financial support for the review, and the role of the funders or sponsors in the review. | Funding statement, p. 10 |
| Competing interests | 26 | Declare any competing interests of review authors. | Conflict of interest statement, p. 11 |
| Availability of data, code and other materials | 27 | Report which of the following are publicly available and where they can be found: template data collection forms; data extracted from included studies; data used for all analyses; analytic code; any other materials used in the review. | Data availability statement, p. 10; Supplementary File 2 |

*From:* Page MJ, McKenzie JE, Bossuyt PM, Boutron I, Hoffmann TC, Mulrow CD, et al. The PRISMA 2020 statement: an updated guideline for reporting systematic reviews. BMJ 2021;372:n71. doi: 10.1136/bmj.n71. This work is licensed under CC BY 4.0. To view a copy of this license, visit <https://creativecommons.org/licenses/by/4.0/>

**PRISMA-S Checklist**

| **Section/topic** | **#** | **Checklist item** | **Location(s) Reported** |
| --- | --- | --- | --- |
| **INFORMATION SOURCES AND METHODS** | | | |
| Database name | 1 | Name each individual database searched, stating the platform for each. | Methods: Literature search strategy, p. 3; Appendix 1 |
| Multi-database searching | 2 | If databases were searched simultaneously on a single platform, state the name of the platform, listing all of the databases searched. | Not applicable; databases were searched separately rather than simultaneously on a single platform. |
| Study registries | 3 | List any study registries searched. | Not applicable; no trial registry was searched as an information source. |
| Online resources and browsing | 4 | Describe any online or print source purposefully searched or browsed (e.g., tables of contents, print conference proceedings, web sites), and how this was done. | Appendix 1 |
| Citation searching | 5 | Indicate whether cited references or citing references were examined, and describe any methods used for locating cited/citing references (e.g., browsing reference lists, using a citation index, setting up email alerts for references citing included studies). | Methods: Literature search strategy, p. 3; Appendix 1 |
| Contacts | 6 | Indicate whether additional studies or data were sought by contacting authors, experts, manufacturers, or others. | Not applicable; study authors or other experts were not contacted for additional studies or data. |
| Other methods | 7 | Describe any additional information sources or search methods used. | Methods: Literature search strategy, p. 3; Appendix 1 |
| **SEARCH STRATEGIES** | | | |
| Full search strategies | 8 | Include the search strategies for each database and information source, copied and pasted exactly as run. | Appendix 1 |
| Limits and restrictions | 9 | Specify that no limits were used, or describe any limits or restrictions applied to a search (e.g., date or time period, language, study design) and provide justification for their use. | Appendix 1 |
| Search filters | 10 | Indicate whether published search filters were used (as originally designed or modified), and if so, cite the filter(s) used. | Database-specific randomized or controlled trial terms were used; no separately published search filter was applied. See Appendix 1. |
| Prior work | 11 | Indicate when search strategies from other literature reviews were adapted or reused for a substantive part or all of the search, citing the previous review(s). | Not applicable; the search strategies were developed for this review and were not adapted or reused from prior literature reviews. |
| Updates | 12 | Report the methods used to update the search(es) (e.g., rerunning searches, email alerts). | Not applicable; no search update was performed after the final search date. |
| Dates of searches | 13 | For each search strategy, provide the date when the last search occurred. | Methods: Literature search strategy, p. 3; Appendix 1 |
| **PEER REVIEW** | | | |
| Peer review | 14 | Describe any search peer review process. | Not applicable; no formal search peer review was conducted. |
| **MANAGING RECORDS** | | | |
| Total Records | 15 | Document the total number of records identified from each database and other information sources. | Results: Study selection, p. 5; Figure 1; Appendix 1 |
| Deduplication | 16 | Describe the processes and any software used to deduplicate records from multiple database searches and other information sources. | Methods: Study selection and data extraction, p. 4; Results: Study selection, p. 5; Figure 1 |
|  |  |  |  |
| PRISMA-S: An Extension to the PRISMA Statement for Reporting Literature Searches in Systematic Reviews | | |  |
| Rethlefsen ML, Kirtley S, Waffenschmidt S, Ayala AP, Moher D, Page MJ, Koffel JB, PRISMA-S Group. | | |  |
|  | | |  |
| Last updated February 27, 2020. | |  |  |

Figure 1. Leave-one-out sensitivity analysis for FOGQ

Figure 2. Leave-one-out sensitivity analysis for UPDRS-III

Figure 3. Leave-one-out sensitivity analysis for BBS

Figure 4. Leave-one-out sensitivity analysis for Mini-BESTest
